# Effect of Scratches on Mechanical Properties and Impermeability of PVC-P Geomembranes

**DOI:** 10.3390/polym17030277

**Published:** 2025-01-22

**Authors:** Xianlei Zhang, Kefan Jiao, Shaoshuai Ma, Yunyun Wu

**Affiliations:** 1 School of Water Conservancy, North China University of Water Resources and Electric Power, Zhengzhou 450046, China; 15939875015@163.com (K.J.); 15839973976@163.com (S.M.); 2 College of Water Conservancy and Hydropower Engineering, Hohai University, Nanjing 210024, China

**Keywords:** geosynthetics, PVC-P GMB, scratches, mechanical properties, impermeability

## Abstract

Plasticized polyvinyl chloride (PVC-P) geomembranes (GMBs) are susceptible to physical scratches due to improper construction in water conservancy projects. Axial tensile tests and permeability tests were carried out to investigate the mechanical properties and impermeability of PVC-P GMBs with scratches under various combinations of scratch angles, lengths, and depths. This was achieved by evaluating the break strength, break elongation, Young’s modulus, and permeability coefficient. The results demonstrated that physical scratches weaken the mechanical properties of PVC-P GMBs, and interactions among the influencing factors were observed. The influence of scratches on the break elongation and break strength outweighed that on Young’s modulus, with scratch depth exerting the most significant effect on the mechanical properties under identical conditions. The scratches on PVC-P GMBs should be minimized in practice, while those without penetrating cracks along the thickness direction and tensile deformation have negligible effects on impermeability. The failure threshold of PVC-P GMBs with scratches was determined, along with the scratch depths, angles, and lengths affecting the operation of the project. This provides a reference for assessing whether PVC-P GMBs with scratches jeopardize the safety of projects.

## 1. Introduction

Plasticized polyvinyl chloride (PVC-P) geomembranes (GMBs) have been widely applied in various applications such as dams, landfills, minerals, and water conveyance tunnels due to their exceptional impermeability, strong adaptability to deformation, convenience for construction, and cost-effectiveness [1,2,3,4]. According to Bulletin 135 by the International Commission on Dams (ICOLD), GMBs have been employed as anti-seepage materials in a total of 167 dams, with PVC-P GMBs being adopted in 86 of them, accounting for approximately 45% of the total. Furthermore, they were applied in 126 small earth-rock dams, with PVC-P GMBs accounting for about 43% of them [5]. The development of pumped storage power stations in China has highlighted the limited deformation adaptability, challenges in underwater repair, and high costs associated with relatively rigid concrete or asphalt concrete slabs of large areas. Consequently, there is growing interest in utilizing GMBs—especially PVC-P GMBs—as anti-seepage materials within reservoir basins. Despite the long service life associated with PVC-P GMBs [6,7], they may be susceptible to physical damages such as wrinkles and scratches caused by transportation, construction, temperature, and cushion particles [8,9,10], which can affect the service life of a project.

Giroud [11] established the bursting and puncture resistance of high-density polyethylene (HDPE) GMB under hydrostatic conditions through laboratory tests. Narejo et al. [12] proposed a method for calculating the mass per unit area of HDPE GMBs with a specified safety factor based on long-term and short-term puncture tests. Xu et al. [13] investigated the mechanical properties of HDPE GMBs with wrinkles and holes and concluded that the presence of holes accelerated the failure of the GMBs. Stark et al. [14] carried out bursting and puncture tests on PVC-P GMBs and explored the relationship between the bursting height, bursting force, and thickness. Nosko and Touze-Foltz [15] illustrated that the presence of holes in GMBs was attributed to the bursting and puncture of the underlying layer. Rowe and Fan [16] examined the effect of the hole geometry on leakage and proposed a theoretical equation for predicting leakage through holes in HDPE GMBs. Giroud and Morel [17] performed a theoretical analysis and obtained the relationships between various parameters such as the height, width, and spacing of wrinkles; the modulus, thickness, density, and coefficient of thermal expansion of a GMB; temperature variations; and GMB-soil friction. Take et al. [18] quantified that 13.9% of the total area of the exposed GMBs were covered by wrinkles using aerial photography and digital image processing. Gao et al. [19] analyzed the mechanism of wrinkles in GMBs around circular structures and concluded that PVC GMBs with higher flexibility can significantly reduce wrinkles. Gudina and Brachman [20] investigated the relationship between the wrinkles of GMBs and subgrade, backfill, and external pressure through experiments. Their findings indicated that these wrinkles have a detrimental effect on the mechanical properties of GMBs. Zhang et al. [21] concluded that wrinkles reduce the mechanical properties of GMBs through experimental analysis. Shu and Pan [22] performed liquid expansion tests on PE and PVC GMBs with scratches, revealing an attenuation in their mechanical properties to a certain extent. Xu et al. [23] investigated the air expansion performance of HDPE GMBs under ring-restrained conditions and determined that defects in GMBs, such as holes, folds, and scratches, decreased both the burst pressure and burst crown height. Cen et al. [24] investigated the local large-strain behaviors of GMBs with circular holes, horizontal cracks, and oblique cracks. The GMB specimens with defects exhibited substantially different tensile behaviors compared with an intact specimen. Cen et al. [25] conducted Califonia Bearing Ratio bursting tests on GMBs with scratches, holes, and cracks, finding that greater scratch depths led to reduced bursting strength for GMBs. Zhang et al. [26] concluded from mechanical tests on different GMBs with scratches that increasing the thickness of GMBs can reduce the performance reduction caused by scratches.

In summary, extensive research has been conducted on the defects of GMBs, with a primary focus on the holes and wrinkles of PE and HDPE GMBs. However, there is a lack of research regarding scratches in PVC-P GMBs. The extent to which scratches affect the mechanical properties and service life of PVC-P GMBs remains unknown from the existing literature. Therefore, it is of significant importance to investigate the mechanical properties and impermeability of PVC-P GMBs with scratches.

This paper aims to investigate the mechanical properties and impermeability of PVC-P GMBs with scratches. The objectives are divided into the following parts: (1) perform axial tensile tests to examine the effect of scratches on the break strength, elongation, and Young’s modulus of PVC-P GMBs with scratches, (2) conduct permeability tests to evaluate the impermeability of PVC-P GMBs with scratches, (3) establish a threshold for the effectiveness of PVC-P GMBs with scratches.

## 2. Materials and Methods

### 2.1. Materials

The commercially available GMB selected in this paper was a 2.0 mm-thick PVC-P GMB made using calendaring technology, with a single roll 45.0 m long and 2.0 m wide. The properties are reported in Table 1 [27,28,29].

### 2.2. Test Methods

#### 2.2.1. Axial Tensile Test

Wide strip specimens were prepared with a width of 200 mm and a gauge length of 100 mm, utilizing a self-developed scratch sampler to make scratches with different angles, lengths, and depths in the center of the specimen. The scratch angle (SA), denoted by α, represents the angle between the direction of the scratch and the axial tensile direction. The angles tested were 0°, 15°, 30°, 45°, 60°, 75°, and 90°. The scratch length ratio (SLR) is defined as the ratio of the scratch length to the width of the specimen, denoted by β. For PVC-P GMBs with a maximum width of 2.0 m, those with scratches where the scratch length ratio (SLR) is β ≥ 50% (i.e., a length no less than 1.0 m) generally require replacement or repair. Therefore, β was set to 10%, 20%, 30%, and 40%, since the width of the specimen was 200 mm and the scratch lengths were 20, 40, 60, and 80 mm, respectively. The scratch depth ratio (SDR), denoted by γ, is defined as the ratio of the scratch depth to the thickness of the specimen. The scratch depth was taken to be 0.5, 1.0, 1.5, and 2.0 mm (indicating penetration), and consequently, γ was 25%, 50%, 75%, and 100%, respectively.

The tensile testing of the geosynthetics was conducted using a CMT-5000 machine (manufactured by Nss (Shenzhen) Laboratory Equipment Co., Ltd., Shenzhen, China) in accordance with ASTM D4885-01 [30] The typical parameters are as follows: a maximum load of 30 kN, a maximum range of 2.1 m, and displacement ranging from 0.2% to 100% of the maximum stroke with an error of 0.5%. The tests were conducted at a temperature of 20 ± 2 °C with a tensile rate of 10 mm/min.

To simulate the scratches on PVC-P GMBs in practice, a series of test schemes was developed, incorporating four SLR sets, four SDR sets, and seven SA sets. Five tensile tests were carried out for each scheme.

#### 2.2.2. Vertical Permeability Test

A GMB vertical permeameter (YT1070, Wenzhou Darong Textile Instrument Co., Ltd., Wenzhou, China) was employed to perform the permeability test. The apparatus comprises a control panel, peristaltic pump, high-pressure chamber, low-pressure chamber (made of transparent organic glass), porous plate, overflow pipe, water storage tank, volumetric flask, and electronic balance. The maximum working pressure of the permeameter is 2.5 MPa, and the effective area of the tested specimen was 200.0 cm^2^.

A scratch sampler was utilized to produce scratches with β values of 10%, 20%, 30%, and 40%. The γ values were 25%, 50%, and 75%. The specimens, with a diameter of 160 mm, had scratch lengths of 16, 32, 48, and 64 mm. The specimen was positioned between the high-pressure chamber and the porous plate. The top of the porous plate was sealed to the low-pressure chamber with a sealing cover featuring a central hole, which connected to one end of the overflow pipe. The other end of the pipe extended into the volumetric flask, where the electronic balance measured the mass of the passing water in real time.

The permeability tests were conducted in accordance with ASTM D5084 [31]. The test pressures were set at 0.4, 0.6, 0.8, 1.0, and 1.2 MPa. Five vertical permeability tests were conducted at each pressure value for a duration of 24 h, during which the electronic balance automatically recorded data every 30 min.

## 3. Results

### 3.1. Results of Axial Tensile Test

**Figure 1:**
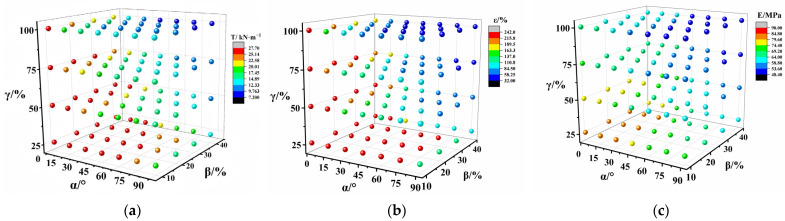
Scatter distribution of EIMPs: (**a**) break strength; (**b**) break elongation; and (**c**) Young’s modulus.

As per ASTM D4885-01, the evaluation indicators for the mechanical properties (EIMPs) were the break strength, break elongation, and Young’s modulus. Figure 1 depicts the scatter distribution of the EIMPs of PVC-P GMBs across all schemes. As described in Section 2.2.1, the dimensions of the wide strip specimen were 200 mm wide with a 100 mm gauge length. The test schemes included four sets of SLRs (10%, 20%, 30%, and 40%), four sets of SDRs (25%, 50%, 75%, and 100%), and seven sets of SAs (0°, 15°, 30°, 45°, 60°, 75°, and 90°). Therefore, a total of 112 (4 × 4 × 7) data points were incorporated into the result analysis.

The EIMPs exhibited varying degrees of degradation when interacting with the SAs, SLRs, and SDRs. Their variation with the SA was found to be correlated with the SLR and SDR. The attenuation of EIMPs under interaction with the SA and SDR followed similar patterns to those with the SA and SLR, with a particular increase in the SDR accelerating the attenuation. When the SA was 60°and the SLR was 20%, as the SDR increased from 25% to 75%, the break strength decreased from 25.14 to 12.33 kN/m, representing a reduction of approximately 51%. The break elongation decreased from 215.8% to 58.25%, corresponding to a reduction of approximately 73%. Additionally, Young’s modulus declined from 69.2 to 53.6 MPa, indicating a decrease of approximately 23%. With the SA and SLR held constant, an increase in SDR resulted in the most pronounced reduction in break elongation, followed by the break strength and finally Young’s modulus.

**Figure 2:**
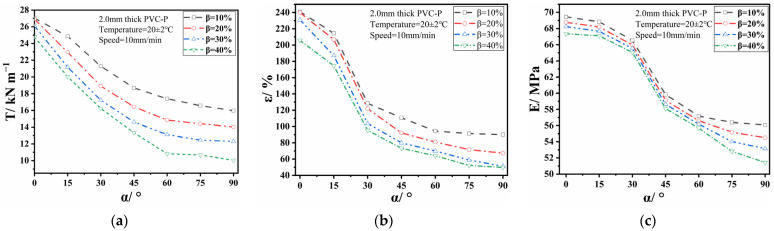
Relationship between EIMPs and SA at γ = 75%: (**a**) break strength; (**b**) break elongation; and (**c**) Young’s modulus.

Figure 2 illustrates the variation in EIMPs with the SA at an SDR of 75%. All EIMP values exhibited a decline. The break strength and break elongation decreased rapidly at α < 60° and slowed down when α > 60°. A rapid decrease in Young’s modulus was observed in the range of 15° < α < 75°, with the highest decay rates in the range from 30° to 45°, while slower rates were observed in other ranges. Young’s modulus quantifies the material’s ability to resist deformation during the elastic deformation stage. A geomembrane with a higher Young’s modulus exhibited greater stiffness, resulting in superior resistance to deformation when subjected to external forces. The rapid decrease in Young’s modulus indicates that the geomembrane was susceptible to greater elastic deformation under the same stress. When holding the SA constant, the EIMP decreased as the SLR increased. The decay rate of the break strength was the highest, followed by the break elongation and then Young’s modulus. Elongation is a crucial parameter for evaluating the adaptability to foundation deformation of PVC-P GMBs. As shown in Figure 2b, the break elongation significantly decreased when the SA was greater than or equal to 45°, whereas no significant difference was observed when the SA was less than 45°. Therefore, it is recommended to limit the SA to within 45° in engineering practice.

**Figure 3:**
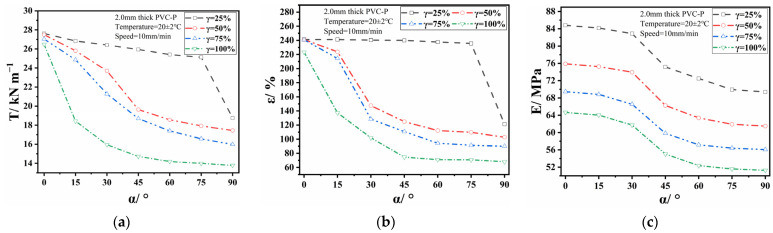
Relationship between EIMPs and SA at β = 10%: (**a**) break strength; (**b**) break elongation; and (**c**) Young’s modulus.

Figure 3 presents the variation in the EIMPs with the SA at an SLR of 10%. It was evident that the EIMPs also showed degradation. Significant decreases in the break strength and break elongation, except for slight decreases under the conditions of γ = 25% and α ≤ 75°, were observed. The attenuation of Young’s modulus with the SA followed consistent patterns across different SLRs, demonstrating a high attenuation rate at 30° < α < 45° and a lower value at other angles. Maintaining a constant SA within 75° resulted in a decreased break strength and elongation as the SDR increased, particularly showing substantial attenuation amplitudes when the SDR ranged from 25% to 75%, indicating a negligible influence when the SDR was less than 25%. Notably, when α ≥ 75°and γ = 25%, there was significant impact on the break strength and elongation. However, the difference in them between γ = 25% and γ > 25% rapidly decreased to a minimum when the SA was 90°. Therefore, to ensure that PVC-P GMBs’ deformation capacity meets a project’s operation requirements, it is recommended that the SDR should not exceed 25% and SA should not exceed 75°. When β = 10%, Young’s modulus displayed a similar downward trend with an increasing SA under different SDRs. However, the difference between adjacent SDRs was small, suggesting insignificant effects from a varying SDR on Young’s modulus.

### 3.2. Results of Vertical Permeability Test

As polymers, GMBs possess extremely small permeation channels, through which water molecules flow at a slow rate. Consequently, the seepage in the membrane was considered laminar flow, consistent with Darcy’s law. The permeability coefficient was determined using the following formula:

(1) $$k_{s}=\frac{V\delta }{A\Delta ht_{s}}\eta$$

where $k_{s}$ is the permeability coefficient of PVC-P GMBs with scratches, V is the passing water volume, δ is the thickness of the specimen, A is the effective permeable area of the specimen, Δh is the head difference, $t_{s}$ is the duration of the water flow V, and η is the correction coefficient of the water temperature.

The permeability coefficients of PVC-P GMBs with scratches were calculated based on the collected passing water volume and Equation (1), and the results are detailed in Table 2.

### 3.3. Preliminary Analysis of Mechanical Properties

#### 3.3.1. Influence of Single Factor

Analysis of variance (ANOVA) was performed to examine the significance of each factor (i.e., SA, SLR, and SDR) on changes in the EIMPs. It is a partitioning of the variation in the data into pieces which can be attributed to different factors and then analyzing whether the factor has a significant impact on the EIMPs.

The SA had 7 levels: SA_1_, SA_2_… SA_7_. All data were regarded as sample values from seven populations (each level was a population). Each population had four SLRs and four SDRs. Therefore, each level had 16 independent data, recorded as $x_{i1}$, $x_{i2}$… $x_{i16}$, where i = 1,2… 7, and the mean values at seven levels were μ_1_, μ_2_,… μ_7_, respectively.

Hypothesis: H_0_: μ_1_ = μ_2_ = …… = μ_7_.

H_1_: μ_1_, μ_2_,… μ_7_ are not completely equal.

The total mean value of the sample $\overset{¯}{x}$ is

(2) $$\overset{¯}{x}=\frac{1}{s\times r}\sum_{i=1}^{r}\sum_{j=1}^{s}x_{ij}$$

where s is the number of independent data and r is the number of levels.

The mean value of the sample at each level ${\overset{¯}{x}}_{i}$ is

(3) $${\overset{¯}{x}}_{i}=\frac{1}{s}\sum_{j=1}^{s}x_{ij}$$

where $x_{ij}$ is the result data of any test, ${\overset{¯}{x}}_{1}$ = μ_1_, ${\overset{¯}{x}}_{2}$ = μ_2_, and ${\overset{¯}{x}}_{7}$ = μ_7_.

The total sum of squares $S_{T}$ can be expressed as

(4) $$S_{T}=\sum_{i=1}^{r}\sum_{j=1}^{s}(x_{ij}-\bar{x}{)}^{2}$$

$S_{T}$ is decomposed into

(5) $$S_{T}=S_{A}+S_{E}$$

where

(6) $$S_{A}=\sum_{i=1}^{r}s({\overset{¯}{x}}_{i}-\overset{¯}{x}{)}^{2}$$

(7) $$S_{E}=\sum_{i=1}^{r}\sum_{j=1}^{s}{\left(x_{ij}-{\bar{x}}_{i}\right)}^{2}$$

$S_{A}$ is the sum of squares for the treatments (or the between-groups sum of squares), and $S_{E}$ is the sum-squared error.

The F distribution is given by

(8) $$F=\frac{S_{A}/(r-1)}{S_{E}/(s-r)}$$

The values of s and r were 16 and 7, respectively. The degrees of freedom for the SA, SLR, and SDR were six, three, and three, respectively. The results of three EIMPs under three single factors were obtained using Equation (8). The impact of each factor on the EIMPs was evaluated using the *F* value. A larger *F* value indicates a greater influence of the factor on the EIMPs. In ANOVA, the typically used *p* value serves as a critical index for assessing the significance of the differences between multiple groups of data. It assists researchers in determining whether there is sufficient evidence to reject the null hypothesis, indicating a significant difference between the groups. In statistical analysis, a significance level (typically set at 0.05) is employed as the threshold for evaluating the *p* value. If the *p* value fell below this threshold, then we rejected the null hypothesis, suggesting a significant difference between the groups. Conversely, if the *p* value exceeded this threshold, then we accepted the null hypothesis, implying no significant difference between the groups. The results of the ANOVA are presented in Table 3, Table 4 and Table 5.

According to the results of the one-way ANOVA, it was found that both the SA and SDR had a significant impact on the EIMPs of the PVC-P GMBs, with *p* values well below 5%. The influence of the SDR on the break strength and Young’s modulus was slightly more pronounced than that of the SA. Additionally, there was no significant difference in the influence of the SDR or SA on the break elongation (*p* values were 2.12 × 10^−11^ and 1.49 × 10^−11^, respectively), whereas the SLR had no significant effect on the EIMPs. However, its influence on the break strength approached the threshold value of 0.05, indicating some level of influence. Consequently, it can be concluded that while the SLR had minimal impact on the EIMPs of the PVC-P GMBs, both the SA and SDR exerted substantial influence.

#### 3.3.2. Comprehensive Influence

Scratches have three elements: the SA, SLR, and SDR. The EIMPs of PVC-P GMBs are influenced by the interaction of these three factors. To analyze the comprehensive influence, α, β, and γ were normalized by Equation (9), denoted as α′, β′, and γ′, respectively:

(9) $$X^{\prime }=\frac{X-X_{min}}{X_{max}-X_{min}}$$

where $X^{\prime }$ is the normalized value of the factors and $X_{min}$ and $X_{max}$ are the minimum and maximum values of the factors, respectively.

A ternary contour phase diagram is a type of barycentric plot which illustrates the proportional relationships among three variables whose sum remains constant. It can reflect the characteristics of data from three different angles. In this paper, a ternary graph is employed to show the difference in the evaluation indicators at different levels of three influencing factors. The ternary contour phase diagram assumes that the relationship between variables is linear.

**Figure 4:**
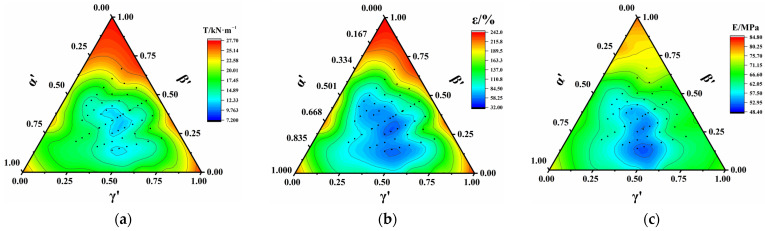
Ternary contour phase diagram of EIMPs: (**a**) break strength; (**b**) break elongation; and (**c**) Young’s modulus.

Figure 4 presents the ternary contour phase diagram of the normalized EIMPs. The EIMPs first decreased and then increased with the increase in β′ at its boundary, inconsistent with the decreasing trend observed in the one-way ANOVA. This suggests that the influence of β′ on the EIMPs relies on the other two factors. A similar phenomenon was observed at the other two boundaries, indicating interaction between the three factors. The EIMPs reached their minimum values in the vicinity of α′ = β′ = γ′ = 0.5, demonstrating the most significant interaction when each factor approached its median value. As for the degree of interaction with the EIMPs, break elongation’s was the highest, followed by Young’s modulus and then the break strength.

Each edge of the ternary graph represents one of the three factors, with values ranging from 0 to 1. At any point along this edge, the other two factors were set to zero and one. Boundary distribution analysis refers to identifying the degree to which EIMPs are influenced by the factors on the edge. The internal points of a ternary graph represent different combinations of three factors. Internal distribution analysis refers to investigating the variation in EIMPs with different combinations of three factors. Based on the boundary distribution, the EIMPs approached high values at the boundaries α′ and β′ while reaching low values at the boundary γ′, indicating a weaker influence from the SA and SLR compared with the SDR, consistent with the one-way ANOVA. Internally distributed data revealed that the EIMPs reached the minimum values at the boundary of γ′, highlighting pronounced influence of the SDR. When γ′ approached 0.5, increasing the SA and decreasing the SLR amplified the attenuation of the EIMPs, which contradicts the one-way ANOVA suggesting reduced attenuation of EIMPs with smaller SLRs. This indicates that reducing the SLR had a weaker effect compared with increasing the SA. When α′ was approximately 0.5, an increase in the SDR and a decrease in the SLR resulted in the initial decrease followed by an increase in the EIMPs. In the region near β′ = 0.5, the increase in the SDR and decrease in the SA led to a similar pattern of an initial decrease followed by an increase in the EIMPs, inconsistent with the findings of the one-way ANOVA. This demonstrates that there exists interaction among the SLR, SDR, and SA, and when these three factors share the same value, they produce the most significant interaction. When γ′ exceeded 0.5, a reduction in the SLR minimized the influence of the SDR on the EIMPs. As β′ surpassed 0.5, diminishing the SA attenuated the effect of the SLR on the EIMPs.

### 3.4. Preliminary Analysis of Permeability

**Figure 5:**
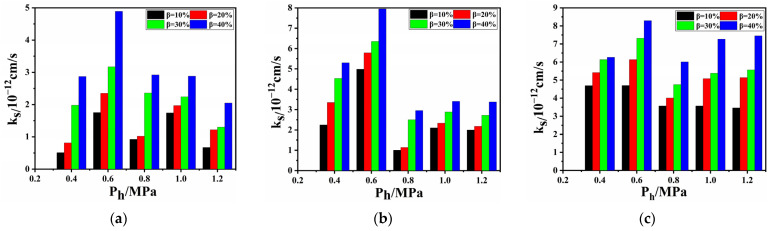
Relationship between permeability coefficient and permeability pressure: (**a**) γ = 25%; (**b**) γ = 50%; and (**c**) γ = 75%.

Figure 5 illustrates the relationship between the permeability coefficient and permeability pressure. The permeability coefficients under four SLRs exhibited a pattern of increase-decrease-increase-decrease with rising permeability pressure. The permeability coefficient was the smallest at 1.2 MPa, with a minimum value of 0.67 × 10^−12^ cm/s at γ = 25%, whereas it was the largest at 0.6 MPa, with a maximum value of 8.29 × 10^−12^ cm/s at γ = 75%. The permeability coefficient increased with the SLR for a constant pressure and also increased with the SDR under the same SLR and pressure. This is attributed to an expanded damaged area caused by the SLR and a reduced effective thickness resulting from the SDR.

## 4. Discussion

### 4.1. Attenuation Rate

The attenuation rate characterizes the effect of scratches on the mechanical properties and impermeability of PVC-P GMBs, defined as the ratio of the difference in the evaluation indicators between the GMB with scratches and the virgin GMB to the evaluation indicators of the virgin GMB. The attenuation rate is expressed as follows:

(10) $$\zeta_{s}=\frac{T_{0}-T}{T_{0}}\times 100\%$$

(11) $$\zeta_{d}=\frac{\varepsilon_{0}-\varepsilon }{\varepsilon_{0}}\times 100\%$$

(12) $$\zeta_{E}=\frac{E_{0}-E}{E_{0}}\times 100\%$$

(13) $$A_{h}=\frac{k_{s}-k_{0}}{k_{0}}\times 100\%$$

where $\zeta_{s}$ is the attenuation rate of the break strength (ARBS), T is the break strength of the GMB with scratches, $T_{0}$ is the break strength of the virgin GMB, $\zeta_{d}$ is the attenuation rate of break elongation (ARBE), ε is the break elongation of the GMB with scratches, $\varepsilon_{0}$ is the break elongation of the virgin GMB, $\zeta_{E}$ is the attenuation rate of Young’s modulus (ARYM), E is the Young’s modulus of the GMB with scratches, $E_{0}$ is the Young’s modulus of the virgin GMB, $A_{h}$ is the attenuation rate of impermeability, and $k_{0}$ is the permeability coefficient of the virgin GMB.

### 4.2. Analysis of ARBS

**Figure 6:**
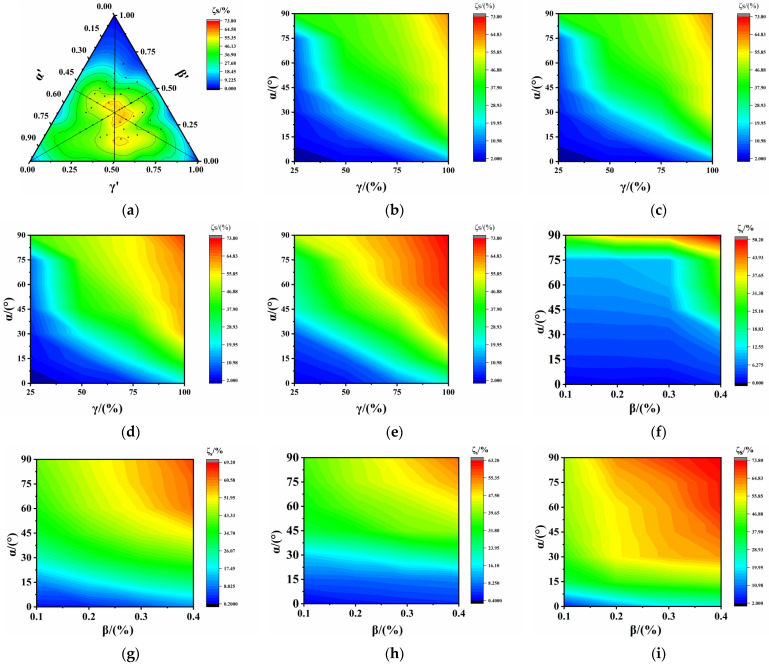
Distribution of ARBS values of PVC-P GMBs with scratches: (**a**) ternary contour phase diagram of ARBS; (**b**) β = 10%; (**c**) β = 20%; (**d**) β = 30%; (**e**) β = 40%; (**f**) γ = 25%; (**g**) γ = 50%; (**h**) γ = 75%; and (**i**) γ = 100%.

Figure 6a illustrates the ternary contour phase diagram of the ARBS using normalized factors. The ARBS was significantly affected in the central region with a maximum value of 60%, demonstrating a significant effect from the SDR.

Figure 6b–e depicts the distribution of the ARBS with the SA and SDR. ARBS values below 10% appeared in the range of α ≤ 7.5°and γ ≤ 45%, whereas ARBS values exceeding 60% were distributed in the range of α > 75°and γ > 90%. No significant variation in the ARBS was observed for β < 40%, indicating no significant interaction between the SA and SDR. However, at β = 40%, ARBS values over 60% extended to the region of α > 30° and γ > 75%, suggesting a substantial interaction between the SA and SDR.

Figure 6f–i presents the distribution of ARBS with the SA and SLR. Comparative analysis demonstrated that the interaction of the SA with the SLR was lowest at γ ≤ 25%, with the maximum ARBS value approaching approximately 35%, a relatively small value. When 50% ≤ γ ≤ 75%, ARBS values below 10% were distributed in a narrower range, whereas ARBS values ranging from 20% to 60% exhibited a broader distribution, indicating a pronounced increase in the ARBS with an increase in the SDR at α > 30°. However, it was worth noting that the ARBS showed no significant correlation with the SLR. The interaction between the SA and SLR significantly intensified with an increasing SDR in the range from 75% to 100%, leading to an increase in the ARBS.

Therefore, the influence of the SLR on the break strength was comparatively less significant than that of the interaction between the SDR and SA. Reducing both the SDR and SA minimized their influence on the break strength, with a greater effectiveness observed in reducing the SDR compared with the SA.

### 4.3. Analysis of ARBE

**Figure 7:**
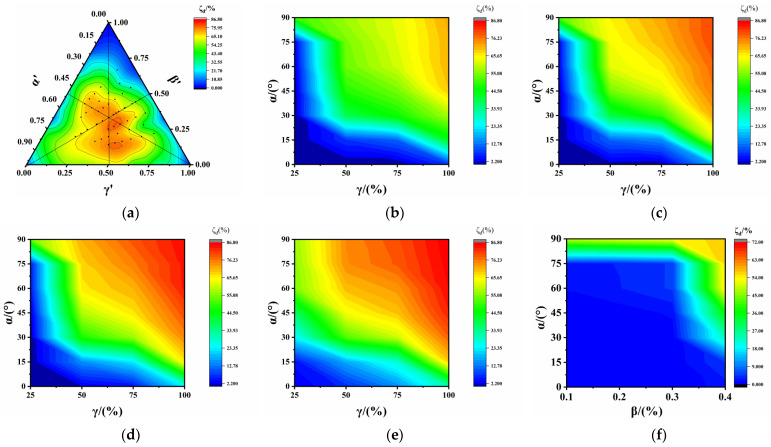

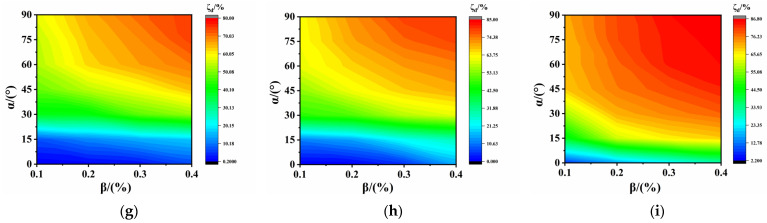
Distribution of ARBE of PVC-P GMBs with scratches: (**a**) ternary contour phase diagram of ARBE; (**b**) β = 10%; (**c**) β = 20%; (**d**) β = 30%; (**e**) β = 40%; (**f**) γ = 25%; (**g**) γ = 50%; (**h**) γ = 75%; and (**i**) γ = 100%.

Figure 7a depicts the ternary contour phase diagram of the ARBE using normalized factors. The ARBE was notably influenced in the central region, reaching a maximum value of 87%, and its variation was affected by the SDR and SA across a larger range.

Figure 7b–e displays the distribution of the ARBE with the SA and SDR. When β ≤ 30%, ARBE values less than 2% occurred in the range of α ≤ 30°and γ ≤ 50%, ARBE values less than 10% were distributed in the range of 30° ≤ α ≤ 80°and 50% ≤ γ ≤ 100%, and ARBE values over 60% appeared in the range of α > 15°and γ > 50%. When comparing Figure 7b and Figure 7c, it was found that the ARBE was not significantly affected by the SLR when its value was smaller than 30%, although it varied slightly in the area with α > 15°and γ > 90%. For β > 30%, the range of ARBE values less than 10% was notably reduced, and that of ARBE values over 60% extended to α > 15° and γ > 60%, indicating the dominance of the SDR in the interaction.

Figure 7f–i illustrates the distribution of ARBE values with the SA and SLR. Comparative analysis indicated that the interaction of the SA and SLR was minimal for γ ≤ 25%, and the ARBE reached a maximum value of approximately 50% in a relatively small range. For 50% ≤ γ ≤ 75%, a narrower range of ARBE values less than 20% and a wider range of ARBE values over 50% were observed. The ARBE increased remarkably with the increase in the SDR at α > 45°, reaching a maximum value of 85%. As the SDR ranged from 75% to 100%, an even broader range of ARBE values over 50% was noted, suggesting that the interaction between the SA and SLR was significantly strengthened with the increasing SDR.

Based on the aforementioned analysis, it was observed that the SLR had no significant influence on the ARBE when β < 30%, α < 15°, and γ < 75%. However, when γ ≥ 75% and α ≥ 15°, any value for the SLR had a notable effect on the ARBE. Therefore, it is imperative to control the SA and SDR to mitigate the ARBE.

### 4.4. Analysis of ARYM

**Figure 8:**
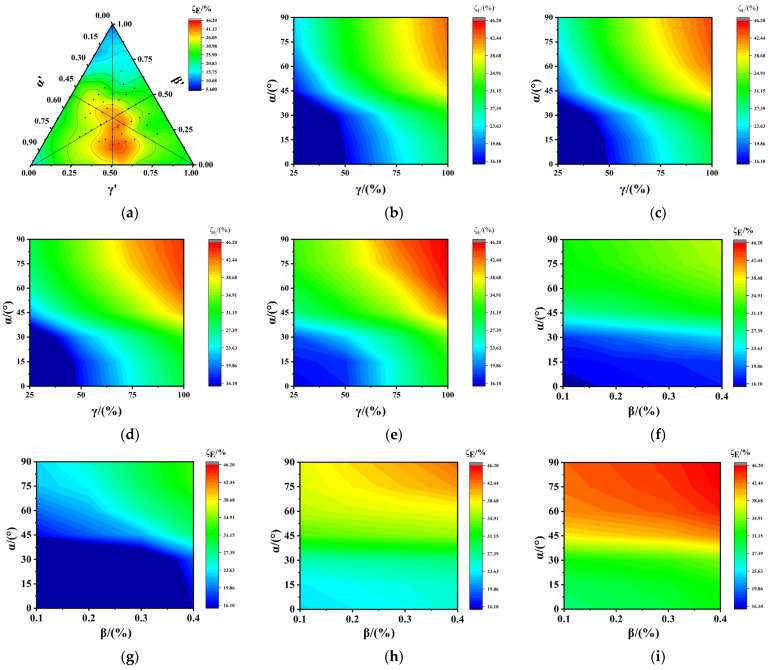
Distribution of ARYM of PVC-P GMBs with scratches: (**a**) ternary contour phase diagram of ARYM; (**b**) β = 10%; (**c**) β = 20%; (**d**) β = 30%; (**e**) β = 40%; (**f**) γ = 25%; (**g**) γ = 50%; (**h**) γ = 75%; and (**i**) γ = 100%.

Figure 8a illustrates the ternary contour phase diagram of the ARYM using normalized factors. The ARYM was significantly affected in the central region with a maximum value of 45%.

The distribution of the ARYM with the SA and SDR is illustrated in Figure 8b–e. When β < 40%, the ARYM was primarily influenced by the interaction of the SA and SDR. ARYM values less than 20% were distributed in the range of α ≤ 42.5°and γ ≤ 75%, whereas ARYM values over 50% were distributed in the range of α > 45°and γ > 90%. From Figure 8c,d, it can be noted that the SLR had a negligible impact on the ARYM, although a somewhat significant effect was evident when γ > 90%. When β ≥ 40%, the distribution of ARYM values over 50% extended to α > 45° and γ > 75%, with little variation in the region where the ARYM values were less than 20%. However, there was a notable reduction in the range where the ARYM values were less than 10%, indicating that the influence of the SLR became more pronounced. Furthermore, ARYM values greater than 50% still occurred in the range where the SDR exceeded 70%, highlighting a significant impact from the SDR at larger values.

Figure 8f–i reports the distribution of ARYM values with the SA and SLR. Comparative analysis revealed that when γ ≤ 50%, regardless of the SDR value, the interaction of the SA and SLR was not statistically significant, and the maximum ARYM value was approximately 30%, indicating a relatively small ARYM. For 50% < γ ≤ 75%, the ARYM exceeded 20% in all cases, with a maximum value of about 42%, the area where the ARYM exceeded 35% expanded, and a noticeable increase in the ARYM with the SDR was observed for α > 75°. At α > 45°, there was a significantly enhanced interaction between the SA and SLR as the SDR increased within the range from 75% to 100%.

After conducting the above analysis, no significant influence from the SLR on the ARYM was observed under the conditions of β < 40%, α < 42.5°, and γ < 75%. However, when β ≥ 40%, γ ≥ 75%, and α ≥ 42.5°, any SLR value had a significant effect on the ARYM. This is primarily attributed to the substantial impact of a larger SDR and SA. Therefore, effective control over the SA and SDR proved to be instrumental in reducing the ARYM.

### 4.5. Attenuation Rate of Impermeability

**Figure 9:**
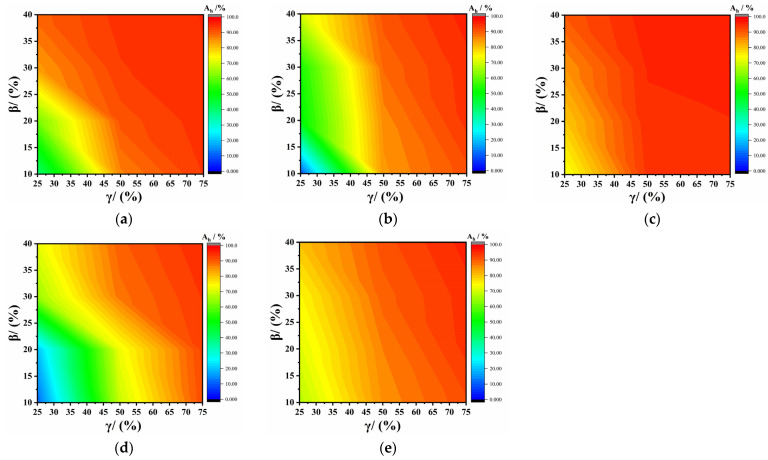
Attenuation rate of impermeability at different pressures: (**a**) 0.4 MPa; (**b**) 0.6 MPa; (**c**) 0.8 MPa; (**d**) 1.0 MPa; and (**e**) 1.2 MPa.

The permeability coefficients of the virgin PVC-P GMBs under 0.4, 0.6, 0.8, 1.0, and 1.2 MPa were 1.55, 0.48, 2.81, 0.57, and 0.61 × 10^−12^ cm/s, respectively. The attenuation rates of impermeability for the PVC-P GMBs with scratches at all test pressures were determined based on Table 2 and Equation (13). Figure 9 presents the distribution of the impermeability attenuation rates for the PVC-P GMBs with scratches under different test pressures. As observed, all attenuation rates did not exceed 100%. The most significant impermeability attenuation occurred at 0.6 MPa, followed by 1.0 MPa, and it was least significant at 0.8 MPa. This is attributed to the crack opening caused by the permeability pressure at the scratch site. Higher pressures may result in smaller crack openings.

The permeability coefficient presented a non-exponential increase at all permeability pressures. PVC-P GMBs without penetrating scratches in the thickness direction and tensile deformation exhibited excellent impermeability, suggesting that implementing appropriate engineering measures to avoid tensile deformation at the scratches can effectively reduce their impact on the impermeability of PVC-P GMBs.

### 4.6. Threshold Value

The impact of the SA, SLR, and SDR on the ARBS, ARBE, and ARYM varied significantly, with an observed interaction among the three factors. The effects caused by different SA, SLR, and SDR combinations were markedly diverse. Therefore, whether scratches affect the safety of projects should be assessed based on the actual engineering.

The lifetime of a GMB can be assessed by the evolution of one or more fundamental characteristics, depending on its nature. For HDPE, different authors have proposed that the service life comes to an end when the design property reduces to 50% of its value [32,33,34]. Koerner et al. [35] predicted the service life of GMBs using a 50% reduction in strength and elongation. Blanco et al. [36] evaluated EPDM GMBs using the elongation at break. As impermeable materials, the adaptability of PVC-P GMBs to deformation of the particle cushion and the dam foundation is crucial, thereby making reductions in the break elongation due to scratches a significant concern. In this paper, a reduction of 50% in the break elongation was considered the failure criterion.

Based on $\zeta_{d}$ ≤ 50%, the scratches with various SA, SLR, and SDR combinations were identified, and they had a negligible influence on the mechanical properties of the PVC-P GMBs. Once scratches not presented in Table 6 occur, those GMBs failing to meet engineering standards should be repaired or replaced. Table 6 provides a reliable basis for promptly assessing whether scratches affect the safety of projects involving the tested PVC-P GMBs.

## 5. Conclusions

This paper presented an investigation into the mechanical properties and impermeability of PVC-P GMBs with scratches. The break strength, elongation, and Young’s modulus were evaluated by conducting axial tensile tests under the SA, SLR, and SDR factors. The impermeability was assessed by performing permeability tests under the combined effect of the SLR and SDR. Based on the experimental results, the following conclusions were reached:

(1) The break strength, break elongation, and Young’s modulus of PVC-P GMBs with scratches all exhibited varying degrees of attenuation. The findings indicated that the significance of these influences primarily relied on the SDR and SA. Therefore, it is essential to monitor the SDR and SA during transportation, loading and unloading, and construction. Furthermore, it is recommended to increase the thickness of PVC-P GMBs to reduce the SDR in harsh environments.
(2) The interaction between the SDR and SA was found to be statistically significant, with a greater impact on the break strength and elongation compared with Young’s modulus. Consequently, engineering quality inspections should examine whether the break elongation and strength of PVC-P GMBs with scratches fulfill the requirements in the specifications.
(3) For PVC-P GMBs with no penetrating scratches in the thickness direction and no tensile deformation in the scratched area, the SLR and SDR had a negligible influence on their impermeability.
(4) The threshold for the effectiveness of PVC-P GMBs with scratches was established, and the scratches not resulting in failure of the PVC-P GMBs were summarized, which provided theoretical references for assessing whether the scratches posed a safety risk to the projects.

This paper provides a method for evaluating the performance of PVC-P GMBs with scratches. The findings provide valuable guidance for GMBs as anti-seepage materials in reservoir basins in engineering design and construction. In this paper, only one PVC-P GMB with scratches was examined for its mechanical properties and impermeability. Due to differences in the composition and content, the results exhibited poor repeatability. In addition, the permeability test did not consider the influence of the tensile deformation and SA. Therefore, further research should investigate the hydraulic performance of PVC-P GMBs with scratches under tensile deformation conditions.

## Figures and Tables

**Table 1:** Properties of tested PVC-P GMBs.

Property	Method	Value(T/L)	Unit
Thickness	ASTM D5199-12 [27]	2.0 ± 0.2	mm
Mass per unit area	ASTM D5261-10 [28]	17.7	kg/m^2^
Break strength Break elongation Yield strength Yield elongation	ASTM D6693/D6693M-04 [29]	9.65/10.10	MPa
		310.10/280.00	%
		2.57/3.39	MPa
		49.85/49.10	%

NOTES: T represents the transverse specimen, and L represents the longitudinal specimen.

**Table 2:** Permeability coefficients of PVC-P GMBs with scratches.

Ph (MPa)	γ = 25%				γ = 50%				γ = 75%			
	β = 10%	β = 20%	β = 30%	β = 40%	β = 10%	β = 20%	β = 30%	β = 40%	β = 10%	β = 20%	β = 30%	β = 40%
0.4	0.51	0.81	1.98	2.87	2.25	3.35	4.53	5.30	4.69	5.42	6.13	6.26
0.6	1.75	2.35	3.17	4.89	4.98	5.79	6.35	7.95	4.70	6.13	7.31	8.29
0.8	0.92	1.02	2.36	2.92	1.00	1.14	2.50	2.95	3.54	4.01	4.75	6.01
1.0	1.74	1.97	2.24	2.88	2.10	2.33	2.88	3.40	3.57	5.08	5.38	7.27
1.2	0.67	1.22	1.30	2.05	2.00	2.18	2.72	3.37	3.46	5.14	5.56	7.45

**Table 3:** ANOVA of break strength.

Source	Degrees of Freedom	Sum of Squares	Mean Square	*F * Value	*p* Value
α	6	2.35	0.39	14.07	1.07 × 10^−11^
β	3	0.36	0.12	2.68	0.051
γ	3	2.07	0.69	23.38	9.79 × 10^−12^

**Table 4:** ANOVA of break elongation.

Source	Degrees of Freedom	Sum of Squares	Mean Square	*F * Value	*p* Value
α	6	4.53	0.75	13.86	1.49 × 10^−11^
β	3	0.50	0.17	1.83	1.45 × 10^−01^
γ	3	3.94	1.31	22.52	2.12 × 10^−11^

**Table 5:** ANOVA of Young’s modulus.

Source	Degrees of Freedom	Sum of Squares	Mean Square	*F* Value	*p* Value
α	6	0.54	0.09	14.71	3.91 × 10^−12^
β	3	0.04	0.01	1.25	2.95 × 10^−01^
γ	3	0.57	0.19	34.14	1.34 × 10^−15^

**Table 6:** Combinations of scratches which did not cause failure of the PVC-P GMBs.

α (°)	β (%)	γ (%)
15	(20, 40)	(25, 75)
30	(10, 20)	(25, 75)
30	(30, 40)	(25, 50)
45	(10, 40)	(25, 50)
60	(10, 30)	25

## Data Availability

The original contributions presented in this study are included in the article. Further inquiries can be directed toward the corresponding authors.
